# Seasonal Pollution Characteristics of Antibiotics on Pig Farms of Different Scales

**DOI:** 10.3390/ijerph19148264

**Published:** 2022-07-06

**Authors:** Delin Du, Jing Zhou, Keqiang Zhang, Suli Zhi

**Affiliations:** 1 Agro-Environmental Protection Institute, Ministry of Agriculture and Rural Affairs, Tianjin 300191, China; ddl199802@163.com; 2 College of Resources and Environment, Northeast Agricultural University, Harbin 150036, China; 3 Guangdong VTR Bio-Tech Co., Ltd., Zhuhai 519060, China; zhoujing0459@163.com; 4 China-UK Agro-Environmental Pollution Prevention and Control Joint Research Centre, Tianjin 300191, China

**Keywords:** veterinary antibiotics, intensive animal farms, seasonal pollution characteristics, ecological risks

## Abstract

Scientific interest in pollution from veterinary antibiotics (VAs) on intensive animal farms has been increasing in recent years. However, limited information is available on the seasonal pollution characteristics and the associated ecological risks of VAs, especially about the different scale farms. Therefore, this study investigated the seasonal pollution status and ecological risks of 42 typical VAs (5 classes) on three different scale pig farms (breeding scales of about 30,000, 1200, and 300 heads, respectively) in Tianjin, China. The results showed that large-scale pig farms usually had the highest antibiotic pollution levels, followed by small-scale pig farms and medium-scale pig farms. Among different seasons, antibiotic contamination was more severe in winter and spring than that in the other seasons. Tetracyclines (TCs) usually had higher proportions (over 51.46%) and the residual concentration detected in manure, and wastewater samples ranged from not detected (ND)-1132.64 mg/kg and ND-1692.50 μg/L, respectively, which all occurred for oxytetracycline (OTC) during winter. For the antibiotic ecological risks in the effluent, we found high-risk level of 12 selected VAs accounted for 58% in spring, and 7 kinds of VAs were selected in the amended soil, but nearly all the antibiotics had no obvious ecological risks except OTC (spring and summer). All these data provided an insight into the seasonal variability and the associated ecological risks of antibiotics on intensive pig farms, which can provide scientific guidance on decreasing antibiotic contamination to enhance environmental security in similar areas.

## 1. Introduction

Animal production is one of the most important sectors of China’s agro-economy. Along with the rapid development of concentrated operations of livestock and poultry, numerous veterinary antibiotics (VAs) have been extensively used in the livestock and poultry industry to treat or prevent bacterial diseases or as growth promoters [1,2]. Furthermore, China’s annual production of antibiotics has been estimated at about 16.2 × 10^4^ tons based on the market sales data in 2013, and more than 7.8 × 10^4^ tons are used for livestock animal breeding each year [3,4,5]. However, 30–90% of these antibiotics would be excreted with animal wastes (manure and wastewater) into the environment due to a combination of poor absorption and incomplete metabolism in vivo [6,7]. Therefore, animal waste has been considered as one of the main reservoirs of antibiotics [8,9,10]. In addition, the annual production of animal waste is very large in China. According to statistics, the amount of manure produced by large-scale livestock farms and poultry breeding in 2015 was 3.834 × 10^9^ tons, including 6.36 × 10^8^ tons of fresh manure and 2.633 × 10^9^ tons of sewage [11]; which could potentially be used as organic fertilizer instead of fertilizer, to promote crop growth and improve soil quality [10,12]. With the utilization of livestock waste in the land, VAs from livestock wastes could be introduced into the soil ecological system, surface water, and groundwater and even generate antibiotic resistance genes (ARGs) and antibiotics resistant bacteria (ARB), which may pose a great potential threat to human public health and cause sustainability concerns [7,9,13,14,15].

According to the literature [16,17], concentrations of various antibiotics in animal wastes were detected that could be up to hundreds of μg/L or mg/kg in different environmental mediums; and the main concern was focused on antibiotic pollution on large-scale farms. For example, two large-scale pig farms in South China were analyzed by Zhang et al. (2018), and the concentrations of oxytetracycline (OTC) and lincomycin (LIN) were higher in pig manure [18]. Zhi et al. (2018) compared the antibiotic pollution in wastewater from large-scale pig farms and dairy farms, which indicated that OTC, chlortetracycline, and doxycycline (DOX) were the main types on pig farms while OTC and LIN were the main on dairy farms [19]. Occasionally, there is a concern about antibiotic pollution on family farms [8]. However, there is no systematic study comparing antibiotic pollution characteristics on different sizes of animal farms. Besides, there are also few reports on the pollution patterns on farms of different sizes in four seasons. Only Pan et al. (2011) gave some results about the variation of VAs in manure on large-scale farms for two seasons [20]. Since the incidence of disease outbreaks on livestock farms varies from season to season, the pattern of antibiotic use should vary with season. There may be seasons when antibiotic use is greater, and the environmental risks are higher. Therefore, understanding the pollution characteristics and environmental risks of antibiotics in different seasons can help people to take measures to prevent pollution in a particular season. Similarly, understanding the characteristics of antibiotic contamination on different scale farms can help to understand the priorities for preventing antibiotic contamination.

Accordingly, in the present study, we investigated three representative pig farms at different scales in Tianjin, China, including small-scale (about 300 heads), medium-scale (about 1200 heads), and large-scale (about 30,000 heads), to take a long-time and detailed seasonal sample (spring, summer, autumn, and winter). These samples covered different environmental mediums (feeds, manure, soils, and wastewater). Totally, 42 commonly used VAs belonging to 5 different classes were selected, containing SAs: sulfonamides antibiotics, TCs: tetracycline antibiotics, QAs: quinolones antibiotics), LAs: β-lactams antibiotics and MAs: macrolides antibiotics. Therefore, the purpose of this paper includes the following aspects: (1) to compare the antibiotic pollution characteristics on different scale pig farms; (2) to analyze the seasonal pollution characteristics of antibiotics on different scale pig farms; (3) to calculate ecological risks of VAs in wastewater and soil fertilized with pig manure; (4) Source analysis of antibiotics in different environmental mediums. This study will provide a more complete understanding of the impact of different seasons and different scales of farms on the pollution characteristics of antibiotics, which will enhance environmental security in similar areas.

## 2. Materials and Methods

### 2.1. Materials and Instrument

The In this study, all antibiotics were widespread used in different scale pig farms in Tianjin, including 42 kinds of VAs belonging to 5 classes. 4 kinds of TCs were included, which consisting of tetracycline (TC, 94.2%), oxytetracycline (OTC, 96.0%), doxycycline (DOX, 98.0%) and demeclocycline (DMC, 92.7%). Sixteen kinds of Sas, which consisting of sulfadimidine (SDMD, 98.0%), sulfamethizole (SMT, 98.0%), sulfamethoxazole (SMX2, 98.0%), sulfabenzamide (SB, 98.0%), sulfameter (SME, 98.0%), sulfamerazine, (SMR, 98.0%), sulfamoxol (SMX1, 98.0%), sulfathiazole (STZ, 98.0%), sulfisoxazole (SIX, 98.0%), sulfadiazine (SDZ, 98.0%), sulfadimidine (SDM, 98.0%), sulfisomidine (SIM, 98.0%), sulfamonomethoxine (SMM, 98.0%), sulfapyridine (SPD, 98.0%), sulfadoxine (SDX, 98.0%), and sulfaquinoxaline (SQX, 98.0%). Fifteen kinds of QAs, which consisting of sparfloxacin (SPA, 98.0%), fleroxacin (FLE, 98.0%), cinoxacin (CIN, 98.0%), enrofloxacin (ENR, 98.0%), ciprofloxacin (CIP, 98.0%), norfloxacin (NOR, 98.0%), ofloxacin (OFL, 98.0%), enoxacin (ENO, 95.8%), sarafloxacin (SAR, 85.8%), difloxacin (DIF, 96.1%), flumequine (FLU, 98.0%), lomefloxacin (LOM, 98.0%), nalidixic acid (NAL, 98.0%), orbifloxacin (ORB, 98.0%), and oxolinic acid (OXO, 98.0%). Two kinds of LAs, which consisting of penicillin G (PENG, 97.1%), and oxacillin (OXA, 98.0%). Five kinds of MAs, which consisting of roxithromycin (RTM, 96.2%), azithromycin (AZI, 98.0%), spiramycin (SPI, 98.0%), clarithromycin (CLA, 98.0%), and tilmicosin (TIL, 80.7%). More details on the standards and reagents for the analyses are provided in Text S1, according to a previous study [19].

### 2.2. Sampling Sites and Sample Collection

Sample sites are located in the Jinghai District of Tianjin, which is in the east of the North China Plains, in the southwest of Tianjin, and in the lower reaches of the Haihe River Basin (38°35′ N–39°4′ N, 116°42′ E–117°12′ E), with an area of 1476 km^2^. The district has a warm temperate continental monsoon climate, which the continental climate is remarkable, with four distinct seasons. The mean temperature in the four seasons of spring, summer, autumn, and winter are 9–20 °C, 21–30 °C, 9–21 °C, and −9–2 °C, respectively, and the mean annual precipitation is 556.7 mm. The basic information about pig farms of different sizes is shown in Table S1.

We selected three representative pig farms and classified them into three categories: small-scale pig farms (breeding scale of about 300 heads), medium-scale pig farms (about 1200 heads), and large-scale pig farms (about 3000 heads). To determine seasonal differences, an equal number of samples of feed, manure, wastewater, and soil were collected from three pig farms in March 2018 (spring), July 2018 (summer), November 2018 (autumn), and January 2019 (winter), respectively. Continuous sampling was conducted for 5 days in each season, and a total of 180 feed samples and 180 manure samples were obtained according to the different pig ages (fatting pigs, piglets, and sows), 60 compost samples were collected from the fecal shed of each farm. One hundred twenty wastewater samples contained 60 influent water samples (IW) and 60 effluent water samples (EW), where the IW and EW mean the wastewater directly from piggery and after a simple storage pool, respectively. One hundred twenty soil samples included 60 reference soil (R-) samples and 60 fertilized soil (F-) samples by livestock manure adjacent to livestock farms. It should be noted that each of the above samples contains three parallel samples. The solid samples were collected by using a multiple-point sampling method, then mixed together to obtain the composite samples and put in clean plastic bags. The liquid samples were collected by samplers and mixed in 500 mL sampling bottles. All samples were numbered and then transported under cooled conditions to the laboratory and stored then stored in the dark at −20 °C.

### 2.3. Sample Pretreatment and Analysis of Antibiotics

For the 180 feed and 120 soil samples, 5.0 g for each sample was analyzed; for manure (180) and composted manure (60) samples, 1.0 g for each sample was analyzed. For the 120 liquid samples, 50 mL was the analysis volume. The detailed pretreatment method and apparatus parameters are described in Texts S2. All the target veterinary antibiotics were analyzed via high-performance liquid chromatography and triple-quadrupole mass spectrometry (HPLC–MS/MS), and the conditions were referred to in a previous report [19].

### 2.4. Ecological Risk

According to previous studies, the potential environmental risks of antibiotics were assessed using the basis of the risk quotient (RQ) schemes, which were calculated by dividing the measured environmental concentrations (MEC) by the predicted no-effect concentrations (PNEC) (Equation (1)) [21]. In this study, maximum MEC was obtained from the occurrence data; PNEC values are estimated by dividing acute half-maximal effective concentrations (EC50), half-maximal lethal concentrations (LC50), or the chronic no observed effect concentration (NOEC) related to toxicological testing with an assessment factor (AF) (Equation (2)) [22,23]. Moreover, only limited information about the PNEC values in soils (PNEC_soil_) has been obtained for calculating RQ value, in which PNEC_soil_ was derived from PNEC values in water (PNEC_water_) by Equation (3), where Kd is the soil-water partition coefficient. The toxicity data of PNECs of antibiotics to the sensitive species were listed in Table S2 (effluent) and Table S3 (soil), respectively. In order to better distinguish the ecological risk levels, the RQ values were generally divided into four risk levels: RQ > 1 (high risk); 0.1 ≤ RQ ≤ 1 (medium risk); 0.01 ≤ RQ < 0.1 (low risk); no risk (RQ < 0.01) [24,25,26].

(1) $$\mathrm{RQ}=\frac{\mathrm{MEC}}{\mathrm{PNEC}}$$

(2) $$\mathrm{PNEC}=\frac{{L(E)C}_{50}}{\mathrm{AF}}$$

(3) $${\mathrm{PNEC}}_{\mathrm{soil}\;}{=\mathrm{PNEC}}_{\mathrm{wastewater}}{\;\times \;K}_{d}$$

## 3. Results and Discussion

### 3.1. Total Antibiotics on Different Scale Pig Farms

**Figure 1:**
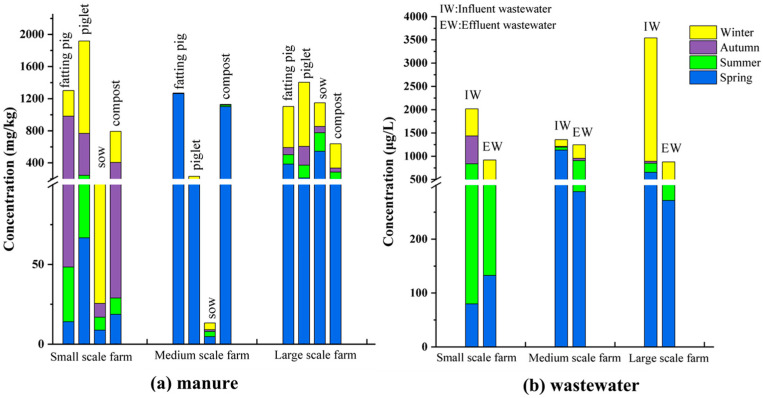
The total average concentrations of antibiotics in manure samples (**a**) and wastewater samples (**b**) on different scale pig farms during the four seasons.

There were large differences in the residual concentration of antibiotics on different pig farms. For example, in sow manure, the total antibiotic concentration was 1147.81 mg/kg for large-scale pig farms, which was much higher than those of small-scale pig farms (191.82 mg/kg) and medium-scale pig farms (13.33 mg/kg) (Figure 1a); in piglet manure, they were 1918.47 mg/kg and 1404.18 mg/kg for small-scale pig farms and large-scale pig farms, respectively, which was also much higher than those of medium-scale farm (231.46 mg/kg) (Figure 1a). Besides, in the influent wastewater (IW), the total antibiotic concentration was 3543.14 μg/L for large-scale pig farms, but it was 2018.27 μg/L and 1356.07 μg/L for small-scale and medium-scale pig farms (Figure 1b). As described above, we can see that large-scale pig farms had the highest overall total concentration of antibiotics, followed by small-scale pig farms and medium-scale pig farms. Although the high prevalence of antibiotics in waste from large-scale farms has been widely reported, the residues of the antibiotics in small-scale farms were also realized. For example, Zhi et al. (2020) investigated 13 family swine farms (<500 pigs) in Dali, Southwest China, and the results showed the total concentrations of 45 VAs could be high to 715.5 mg/kg in manure and 12,704.4 μg/L in wastewater [8]. Chen et al. (2012) also indicated that the smaller units showed higher contamination levels of some antibiotics [27]. In other countries, one similar previous study carried out in Thailand showed that the total concentration of 17 VAs for small-scale pig farm effluents (24.90 μg/L) was higher than that of medium-scale farms (13.01 μg/L) [28]. These observations may be attributed to the different usage modes and doses of antibiotics in pig farms in different countries and regions.

Moreover, in this study, we found that the residues of antibiotics in piglet and fattening pig manure were higher than those in sow manure (Figure 1a), and the results were consistent with the effect of swine types on antibiotic residues in China reported by Hou et al. (2015), which also indicated that more antibiotics were used for piglets and fattening pigs to enhance feed efficiency, reduce the incidence of disease and visibly shorten their growth time [29,30]. What is more, the absorptive capacity for antibiotics in different growth stages of pigs as well as the farm management, such as waste storage, breeding performances, and the usage habits of antibiotics, may also affect the antibiotic residues in feces and wastewater on different pig farms [31,32].

### 3.2. Seasonal Variance of Antibiotics Concentration on Different Scale Pig Farms

**Figure 2:**
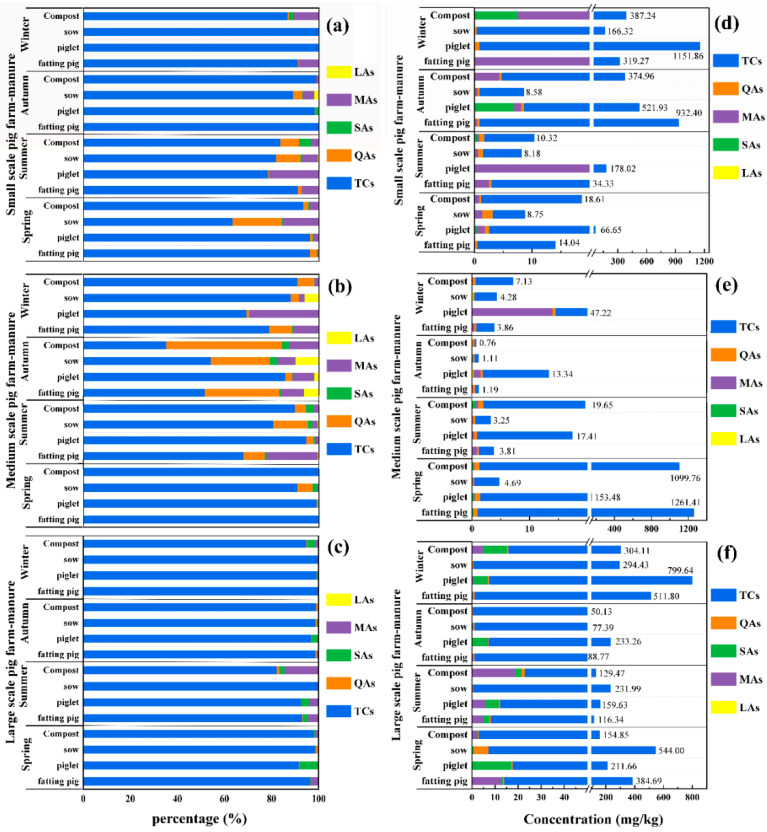
The proportions and concentrations of five classes of antibiotics detected in manure on different scale pig farms during the four seasons. ((**a**): small farm for VAs percentage; (**b**): medium farm for VAs percentage; (**c**): large farm for VAs percentage; (**d**): small farm for VAs concentration; (**e**): medium farm for VAs concentration; (**f**): large farm for VAs concentration).

**Figure 3:**
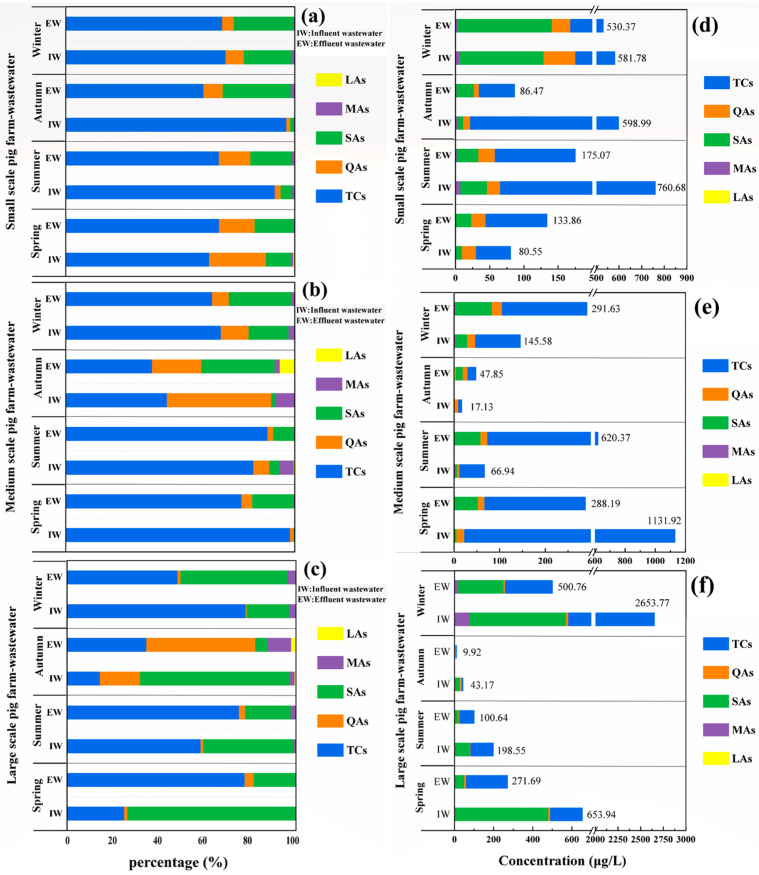
The proportions and concentrations of the five classes of antibiotics detected in wastewater on different scale farms during four seasons. ((**a**): small farm for VAs percentage; (**b**): medium farm for VAs percentage; (**c**): large farm for VAs percentage; (**d**): small farm for VAs concentration; (**e**): medium farm for VAs concentration; (**f**): large farm for VAs concentration).

This section will focus on the residual levels of 5 classes of antibiotics during different seasons. Figure 2 and Figure 3 show the “residual concentration” and “percentage” of different VAs during four seasons in manure and wastewater, respectively. Among the five selected VAs classes, the total concentration of TCs was the highest both in manure and wastewater during four seasons, while the other four classes were different with seasonal variation.

In the large-scale pig farm (breeding scale about 30,000 heads), the seasonal pollution level of antibiotics was the lowest in autumn and the highest in winter. As shown in Figure 2f, the total average concentrations of the selected antibiotics in manure of sows, piglets, fatting pigs and their compost were much higher in winter than that in autumn (294.43 vs. 77.39 mg/kg; 799.64 vs. 233.26 mg/kg; 511.80 vs. 88.77 mg/kg; 304.11 vs. 50.13 mg/kg, respectively). As for the five classes of antibiotics, the percentages in winter were TCs (98.67%) > SAs (0.90%) > MAs (0.32%) > QAs (0.11%) > LAs (0.00%) (Figure 2c); in autumn, TCs (97.72%) > SAs (1.59%) > MAs (0.34%) > QAs (0.29%) > LAs (0.05%) (Figure 2c). Similar to manure, as shown in Figure 3f, the total average concentrations in IW and EW were much higher in winter than that in autumn (2653.77 vs. 43.17 μg/kg; 500.76 vs. 9.92 μg/kg, respectively). However, for the percentages of the five classes of antibiotics, TCs (63.24%) > SAs (32.67%) > MAs (3.34%) > QAs (0.75%) >LAs (0.00%) during winter (Figure 3c); in autumn SAs (36.67%) > QAs (32.39%) > TCs (22.30%) > MAs (7.23%) > LAs (1.42%) (Figure 3c). Notably, the proportion of SAs even account for up to 73.44% in the influent samples during spring. However, this was not surprising because the weak adsorption of SAs in manure and their persistent trend in wastewater samples [14].

In the medium-scale pig farm (about 1200 heads), the seasonal pollution level of antibiotics was the lowest in autumn and the highest in spring. In Figure 2e, the total average concentrations of antibiotics in manure of sow, piglet and fatting pig and their compost were higher in spring than that in autumn (4.69 vs. 1.11 mg/kg; 153.48 vs. 13.34 mg/kg; 1261.41 vs. 1.19 mg/kg; 1099.76 vs. 0.76 mg/kg, respectively), for different classes of antibiotics, the percentages during spring were TCs (99.83%) > QAs (0.11%) > SAs (0.04%) > MAs (0.01%) = LAs (0.01%); in autumn, TCs (78.73%) > MAs (9.42%) > QAs (8.55%) > LAs (2.72%) > SAs (0.57%) (Figure 2b). In swine wastewater of IW and EW, the concentrations of antibiotics were much higher in spring than that in autumn (1131.92 vs. 17.13 μg/kg; 288.19 vs. 47.85 μg/kg, respectively) (Figure 3e), and in Figure 3b, during spring TCs (87.42%) > SAs (9.27%) > QAs (3.07%) > MAs (0.16%) > LAs (0.07%); in autumn, TCs (42.79%) > QAs (32.13%) > SAs (16.15%) > MAs (5.66%) > LAs (3.27%).

In the small-scale pig farm (about 300 heads), the seasonal pollution level of antibiotics was the lowest in spring and the highest in winter. As shown in Figure 2a, the total average concentrations of antibiotics in different kinds of manure were higher in winter than that in spring (166.32 vs. 8.75 mg/kg; 1151.86 vs. 66.65 mg/kg; 319.27 vs. 14.04 mg/kg; 387.24 vs. 18.61 mg/kg, respectively), the percentages of different classes of antibiotics during winter were TCs (95.96%) > MAs (3.46%) > SAs (0.39%) > QAs (0.19%) > LAs (0.00%); in spring, TCs (93.02%) > MAs (3.25%) > QAs (3.05%) > SAs (0.67%) > LAs (0.01%) (Figure 2d). For wastewater including IW and EW, in winter and spring were 581.78 vs. 80.55 mg/kg and 530.37 vs. 133,86 mg/kg, respectively (Figure 3d), the percentages of different classes of antibiotics during winter were TCs (69.15%) > SAs (23.57%) > QAs (6.46%) > MAs (0.82%) > LAs (0.00%); in spring, TCs (65.37%) > QAs (20.23%) > SAs (13.33%) > MAs (0.80%) > LAs (0.27%) (Figure 3a).

To summarize, in the three pig farms, the seasonal pollution of antibiotics was more serious in winter and spring than that in summer and autumn, and the seasonal variation of antibiotics in this study was generally similar to the residual characteristics of 14 VAs in manure collected from a large-scale pig farm (25,000 pigs) during winter and summer in Jiangsu Province, China [33]. This may be due to the outbreaks of diseases that are usually higher in spring and winter, such as influenza and other respiratory diseases caused by low outdoor temperatures, especially in winter [7,27]. Besides, LAs existed only in autumn and accounted for 0.05% of the total average antibiotic concentration, which implied less use of this type of antibiotic in swine breeding.

For different classes of antibiotics, TCs were usually the dominant antibiotics found in pig waste (manure and wastewater), followed by SAs and QAs. They account for more than 90% of the total concentrations on different pig farms. Based on previous studies [34,35], these three classes of antibiotics are commonly used in the animal breeding industry because of their low price and broad-spectrum [32,34,35,36]. In China, TCs had been used in pig husbandry for more than 50 years, which were commonly used to increase the growth of animals and prevent disease during different growing periods by in-feed antibiotics or injection [3,37,38,39]. In addition, the weak regulation of antibiotics and lower prices of TCs were also important reasons why they are used in breeding frequently (http://www.syyl.org/index.asp, accessed on 13 June 2018, in Chinese). This is why TCs usually has high levels in pig farm waste.

### 3.3. Single Antibiotic Concentration during Different Seasons

Although seasonal pollution characteristics of different classes were analyzed, it was also meaningful to analyze the seasonal variation of a single antibiotic. The concentrations of 15 major antibiotics during the four seasons were shown in Table 1 (manure) and Table 2 (wastewater). The other 27 antibiotic concentrations are shown in Tables S4 and S5. Overall, TCs class (OTC, TC, DXC, and DMC) were dominant types both in manure and wastewater samples, with high concentrations which were approximately 20 times higher than that of others. For example, Table 1 showed that OTC had the highest concentration in manure among all 42 VAs, with a concentration of 1132.64 mg/kg (mean: 313.81 mg/kg) in winter and 1234.79 mg/kg (mean: 140.19 mg/kg) in spring, respectively. In addition, the maximum concentrations of TC (27.69 mg/kg) and DMC (11.61 mg/kg) were also detected in winter (Table 1). In wastewater, OTC had the highest concentration with 1692.50 μg/L (mean: 432.27 μg/L) in winter, followed by spring of 1071.91 μg/L (mean: 292.56 μg/L). However, the maximum concentrations of TC (75.86 μg/L) and DMC (9.61 μg/L) were detected in summer (Table 2). Their residual concentrations in pig farm waste were higher in spring and winter than in other seasons. This result is similar to that in another study [33], although the concentration of TCs in manure was several times lower than that in this study [33,36].

As for SAs, SDMD, SMM, and SME were the dominant types, and the maximum concentrations reached up to 10.03 mg/kg (autumn), 6.69 mg/kg (spring), and 0.35 mg/kg (winter), respectively (Table 1). In wastewater (Table 2), SDMD was detected highest concentration among all SAs compounds, reaching up to 479.43 μg/L (mean: 125.38 μg/L) in winter and 464.44 μg/L (mean: 92.42 μg/L) in spring; followed by the SMM, with the maximum concentration of 123.33 μg/L (mean: 78.96 μg/L) in winter. In this study, some types of SAs were quantitatively detected and showed different seasonal variations. This result exposed that the usage and dosage of SAs varied greatly during different seasons. SAs are specific pharmaceuticals which usually added to feed in the swine industry for curing or preventing certain diseases [40]. In addition, the residual concentration of SMT and SMX in manure (Tianjin, North China) measured by Hu et al. (2010) and Hu et al. (2008) were 3.30–24.80 mg/kg and 0.23–5.70 mg/kg, respectively, which were greater than the results of this study [41,42]. Besides, Zhou et al. (2020) also detected high level of SAs in swine manure from four large-scaled swine farms in Jiangsu Province of China; and the results showed that: SPD (4.96 mg/kg) > SMM (3.49 mg/kg) > SMX2 (0.49 mg/kg) > SDZ (0.14 mg/kg) [43].

Among QAs, OFL, CIP and ENO maximum concentrations were all observed in spring, which were 5.87 mg/kg (mean: 0.67 mg/kg), 1.73 mg/kg (mean: 0.28 mg/kg), and 0.11 mg/kg (mean: 0.09 mg/kg), respectively (Table 1 and Table S4). In addition, the maximum residual concentrations of SAR, OFL, FLU, and LOM exceeded 5.00 μg/L in wastewater with values of 27.32 μg/L (winter), 11.00 μg/L (winter), 8.83 μg/L (winter), and 6.72 μg/L (summer), respectively (Table 2). The results showed that higher mean concentrations of some QAs compounds such as OFL, CIP, ENO, and SAR were detected in pig farm waste during spring and winter, which may be due to their higher usage to prevent higher morbidity in these two seasons [14,27]. In addition, the maximum and mean concentrations of NOR, ENR, ORB, CIP, and ORL in the manure were equivalent to the previous result from other swine farms located in eastern and southwestern regions of China [8,43].

For the other classes of antibiotics, the maximum residual concentrations of PENG in manure and wastewater were 0.26 mg/kg (autumn) and 3.09 μg/L (autumn), respectively (Table 1 and Table 2). While another report showed that PENG had a high concentration of ND-3145.18 μg/L in wastewater, this may be due to the different antibiotic use habits, waste treatment facilities, animal types, and so on [8]. Among MAs, AZI and TIL were the dominant types, and the maximum concentrations detected in manure (Table 1) showed higher values with 9.92 mg/kg (spring) and 37.81 mg/kg (summer), respectively. In wastewater, for both AZI and TIL, their residual concentrations were higher in winter than in other seasons (Table 2). The mean concentration levels of other antibiotic contaminants were all below 0.04 mg/kg. In fact, AZI and TIL were often used as drugs to treat pig diseases, which had become emerging contaminants for humans [44]. But the studies involving the seasonal variation of MAs in farm waste are insufficient and need more attention.

From the above, different kinds of antibiotics showed different seasonal pollution characteristics, but most antibiotic compounds are usually detected with higher residual levels during winter and spring, which was in accordance with the results in Section 3.2.

### 3.4. Environmental Risk Assessment for Antibiotics

The high levels of antibiotics were frequently found in the surrounding environment (effluent and soil), especially in areas that had large numbers of livestock farms, which can pose some ecological risks associated with the use of manure as fertilizers in agricultural soils [45]. Therefore, it is of great significance to understand the current status as well as the environmental risks of antibiotic pollution.

**Figure 4:**
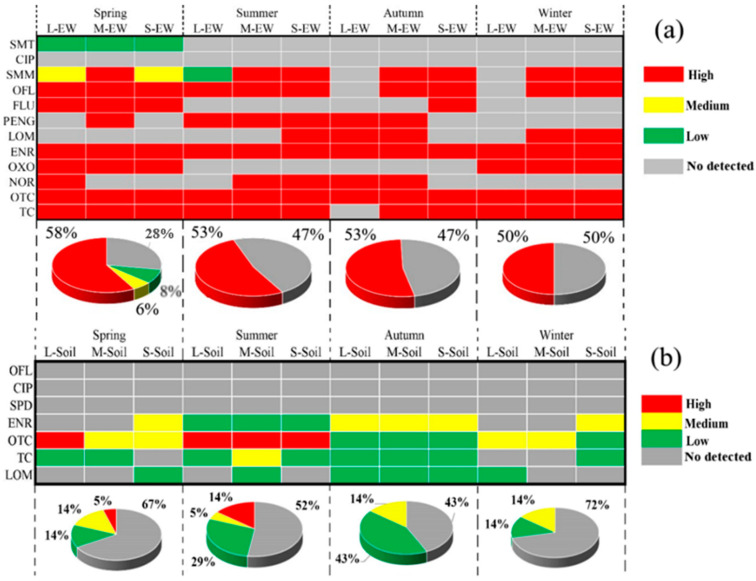
Risk assessment of antibiotics in effluent wastewater (**a**) and soil (**b**).

Comparing the effluent samples collected during different seasons, the ecological risks posed by some antibiotics to aquatic organisms were high in wastewater. It was surprising to find that OFL, FLU, ENR, OXO, OTC, and TC all showed high-risk levels during spring (Figure 4a). What is more, for some samples collected in other seasons, these antibiotics also showed high ecological risk (Figure 4a), which was related to the abundance of antibiotics in the effluent of the pig farms. For soil, OTC also showed high-risk levels during spring and summer, respectively (Figure 4b). ENR posed a medium risk to aquatic organisms during spring, summer, and winter, respectively. RQs of TC were lower in all seasons except summer; however, OTC still showed medium risk levels in autumn and winter. This may be due to TCs potentially posing a risk even at very low concentrations. Some previous studies also conducted an ecological risk assessment of these selected antibiotics. For example, Wei et al. (2019) analyzed the antibiotic pollution in vegetable farm soils fertilized with livestock excreta, which indicated that OTC indeed posed a high risk to the soil environment [46]. Moreover, some reports also showed that ENR and CIP posed a severe ecological risk to the soil environment (Li et al., 2015; Zhao et al., 2019) [47,48]. Besides, the RQs were lower in soil than that of wastewater samples, indicating that the composting technology can effectively reduce the ecological risk posed by antibiotics [49,50]. Moreover, antibiotics also can be degraded in soil due to microbial degradation, soil adsorption, photolysis, and so on. However, Li et al. (2015) found ENR, CIP, and OTC could exhibit a high risk to soil organisms in greenhouse soils during spring and summer [47].

According to the ecological risk levels in the surrounding environment of the pig farms (Figure 4), the results showed that samples collected from spring were more inclined to pose a high risk. However, Ho et al. (2012) and Wei et al. (2019) found there are some limitations to assessing the environmental risk in the soils by using the RQs method; this may be because we are not able to collect all the toxicity data and the solid-water partition coefficient values in the literature [46,51]. Moreover, synergistic toxicity between different antibiotics is still lacking. Therefore, synergistic risk assessments associated with different antibiotics from pig waste should be a hot topic in the future.

### 3.5. Influencing Factors of Main Antibiotics in Livestock Manure and Wastewater

**Figure 5:**
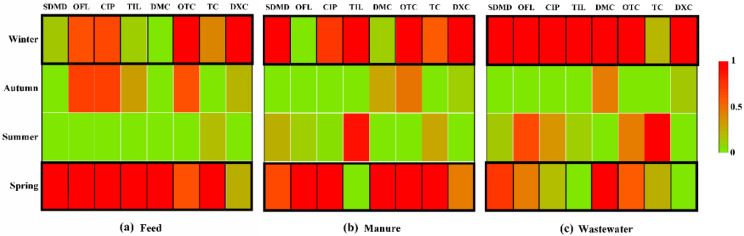
Detection concentration characteristics of antibiotics in waste (manure (**b**) and wastewater (**c**)) and the corresponding feed (**a**).

We examined in detail the potential effects of food sources on antibiotic residues in livestock waste. From Figure 5, especially where were marked with black borders, the trend of most antibiotics in manure was similar to that in pig feed during spring and winter (Figure 5a,b)). In addition, wastewater also presented some similar rules during spring and winter (Figure 5a,c)), which corresponded to high concentrations of antibiotics in pig feed. However, compared with the similar residual laws between feed and feces, the similarity in wastewater was not obvious, which may be due to too many impurities in wastewater and various effects (decomposition or enrichment) of antibiotics. One previous study has found that most antibiotics in the effluent were residual compounds from the pig feed. Shen et al. (2014) also found that OTC concentrations in manure from piglets were positively correlated to those in their feeds [52]. Similar results were also reported in a previous report [8]. In order to identify the influencing factors of antibiotics in pig manure samples with relatively high antibiotic concentrations, we further analyzed the statistical correlation between influencing factors (seasonal changes, antibiotics in feed, and pig farm size) and antibiotic residues in pig manure and wastewater (Figure S1). The results showed that the antibiotic concentrations in feed had an extremely significant correlation (*p* < 0.01) with the antibiotic concentrations in pig wastes. This is also consistent with the conclusion in Figure 5. Farm scale and season also had a significant correlation with a few antibiotics, e.g., SDMD was more likely significantly affected by farm scales; TIL and DXC may be affected by different seasons.

In conclusion, the high concentrations of some antibiotics in feed precisely explained why their concentrations were relatively high in swine manure and wastewater. However, the relatively high levels of antibiotics in manure not only from animal feeds but may also from other administration routes, such as adding antibiotics to drinking water, injection, or special oral treatment [27]. In addition, antibiotics in pig wastes were also affected by many other factors (antibiotic type, usage mode and dose, livestock age and intestinal flora, etc.) [8,19]. The distribution coefficients, transformation, adsorption, and degradation of antibiotics in the environment were important for residual antibiotic levels, which is why wastewater concentration has less correlation with feed concentration than manure had [19,27,31].

## 4. Conclusions

In order to explore the seasonal pollution characteristics of antibiotics and their ecological risk, we selected three different scale pig farms (about 30,000, 1200, and 300 head farms) in Tianjin city, Northern China, and conducted continuous seasonal sampling (feed, manure, wastewater, and soil). The results showed that the exposure concentrations of antibiotics in the three different pig farms decreased in the following order: large-scale farm > small-scale farm > medium-scale farm. The seasonal pollution levels of antibiotics in spring and winter were higher than that in summer and autumn among the three farms. Among the different classes of VAs, TCs was the most prominent type in all manure and wastewater samples, which had high concentrations and proportions compared to the other four classes of VAs (QAs, SAs, LAs, MAs). Traceability of eight commonly used antibiotics with high concentrations had been found to be related to the use of antibiotics added to feed in pig farms, and seasonal changes and the size of pig farms also affected the residual concentrations of antibiotics in livestock waste. Ecological risk assessment based on the RQs revealed that 10 out of all 12 antibiotics presented high ecological risk in effluent samples, especially in spring. Seven kinds of antibiotics were also assessed for ecological risks in the soil, but no antibiotic showed a high-risk level in soil except OTC. Based on the above results, especially in winter and spring, the concentrations of VAs and the associated ecological risk to the surrounding environment should be paid more attention to.

## Figures and Tables

**Table 1:** The main individual antibiotic concentration in manure in spring, summer, autumn, and winter (mg/kg).

Antibiotics		Spring (*n* = 60)			Summer (*n* = 60)			Autumn (*n* = 60)			Winter (*n* = 60)		
Class	Type	Min	Max	Mean	Min	Max	Mean	Min	Max	Mean	Min	Max	Mean
TCs	OTC	ND	1234.79	140.19	ND	226.19	63.81	ND	925.15	186.65	ND	1132.64	313.81
	TC	ND	26.61	8.43	ND	13.74	3.88	ND	8.63	2.13	ND	27.69	5.86
	DXC	ND	10.95	1.90	ND	1.60	0.27	ND	5.48	0.75	ND	11.61	3.53
	DMC	ND	2.46	0.72	ND	1.59	0.27	ND	0.48	0.12	ND	0.87	0.20
QAs	OFL	ND	5.87	0.67	ND	0.95	0.11	ND	0.04	0.04	ND	0.05	0.04
	LOM	ND	0.07	0.04	ND	0.03	0.02	ND	0.03	0.03	ND	0.07	0.04
	SAR	ND	0.04	0.04	ND	0.02	0.01	ND	0.04	0.04	ND	0.05	0.04
	CIP	ND	1.73	0.28	ND	0.10	0.04	ND	0.04	0.04	ND	1.33	0.18
	FLU	ND	0.04	0.03	ND	0.01	0.00	ND	0.03	0.02	ND	0.03	0.02
SAs	SMM	ND	0.24	0.06	ND	0.19	0.04	ND	6.69	0.84	ND	1.37	0.13
	SDMD	ND	10.06	1.69	ND	5.65	0.97	ND	6.55	0.63	ND	10.06	2.06
	SME	ND	0.25	0.05	ND	0.23	0.04	ND	0.19	0.02	ND	0.35	0.08
MAs	AZI	ND	9.92	0.96	ND	0.14	0.03	ND	0.08	0.03	ND	7.64	0.76
	TIL	ND	2.97	0.71	ND	37.81	6.04	ND	4.28	0.71	ND	34.32	6.78
LAs	PENG	ND	0.11	0.11	ND	0.03	0.02	ND	0.26	0.13	ND	0.26	0.26

ND: not detected; *n*: number of samples analyzed; Max: maximum concentration (mg/kg); Min: minimum concentration (mg/kg); 0.00: <0.005 mg/kg. The concentrations of the other 27 antibiotics are shown in Table S4.

**Table 2:** The main individual antibiotic concentration in wastewater in spring, summer, autumn, and winter (μg/L).

Antibiotics		Spring (*n* = 30)			Summer (*n* = 30)			Autumn (*n* = 30)			Winter (*n* = 30)		
Class	Type	Min	Max	Mean	Min	Max	Mean	Min	Max	Mean	Min	Max	Mean
TCs	OTC	ND	1071.91	292.56	ND	600.04	238.63	ND	477.54	89.79	ND	1692.50	432.27
	TC	ND	29.52	8.97	ND	75.86	24.24	ND	24.42	6.12	ND	24.37	8.74
	DXC	ND	12.08	4.47	ND	ND	ND	ND	73.59	21.64	ND	352.51	118.47
	DMC	ND	2.71	1.89	ND	ND	ND	ND	1.94	1.34	ND	2.48	1.71
QAs	OFL	ND	6.40	3.09	ND	8.49	4.74	ND	2.01	1.42	ND	11.00	6.16
	LOM	ND	1.71	1.61	ND	6.92	3.81	ND	ND	ND	ND	ND	ND
	SAR	ND	2.79	2.09	ND	5.11	3.21	ND	1.35	1.06	ND	27.32	6.92
	CIP	ND	0.78	0.75	ND	ND	ND	ND	0.50	0.50	ND	0.56	0.55
	FLU	ND	2.27	1.52	ND	2.42	1.50	ND	1.28	1.11	ND	8.83	3.34
SAs	SMM	ND	22.33	5.89	ND	15.88	6.04	ND	21.74	13.57	ND	123.33	78.96
	SDMD	ND	464.44	92.42	ND	78.36	23.95	ND	27.36	7.17	ND	479.43	125.38
	SME	ND	8.06	2.13	ND	20.05	7.42	ND	0.96	0.56	ND	14.67	5.63
MAs	AZI	ND	0.28	0.27	ND	1.71	0.88	ND	0.62	0.52	ND	10.5	2.55
	TIL	ND	0.54	0.41	ND	4.30	2.21	ND	1.86	0.80	ND	65.33	15.49
LAs	PENG	ND	0.25	0.21	ND	0.78	0.38	ND	3.09	1.16	ND	ND	ND

ND: not detected; *n*: number of samples analyzed; Max: maximum concentration (μg/L); Min: minimum concentration (μg/L). The concentrations of the other 27 antibiotics are shown in Table S5.

## Data Availability

Data sharing is not applicable to this article.

## Supplementary Materials

The following supporting information can be downloaded at: https://www.mdpi.com/article/10.3390/ijerph19148264/s1, Texts S1: Chemicals and materials; Texts S2: Sample preparation for analysis of antibiotic content; Table S1: Basic information of pig farms of different sizes; Table S2: Toxicity data of antibiotics corresponding to sensitive species (wastewater); Table S3: Toxicity data of antibiotics corresponding to sensitive species (soil); Table S4: The other 27 antibiotics concentration in manure in spring, summer, autumn, and winter (mg/kg); Table S5: The other 27 antibiotics concentration in wastewater in spring, summer, autumn, and winter (μg/L); Figure S1: The potential effects of seasonal variation, feed and pig farm size on antibiotic residues in pig manure and wastewater. References [43,53,54,55,56,57,58,59] are cited in the supplementary materials.
