# Supplementary material for: Seasonal Pollution Characteristics of Antibiotics on Pig Farms of Different Scales
Source: Int J Environ Res Public Health. 2022 Jul 6;19(14):8264. doi: 10.3390/ijerph19148264 (PMC9320919; doi:10.3390/ijerph19148264)
Supplement: Supplementary file 1 [file ijerph-19-08264-s001.zip › ijerph-1749888-supplementary.pdf]

Supplementary materials for Seasonal Pollution Characteristics of Antibiotics on Pig Farms of Different Scales

Delin Du [1,2], Jing Zhou [3], Keqiang Zhang [1,2,*] and Suli Zhi [1,4,*]

[1] Agro-Environmental Protection Institute, Ministry of Agriculture and Rural Affairs, Tianjin 300191, China; ddl199802@163.com
[2] College of Resources and Environment, Northeast Agricultural University, Harbin 150036, China
[3] Guangdong VTR Bio-Tech Co., Ltd., Zhuhai 519060, China; zhoujing0459@163.com
[4] China-UK Agro-Environmental Pollution Prevention and Control Joint Research Centre,
Tianjin 300191, China
* Correspondence: keqiangzhang68@163.com (K.Z.), zhisuli87@163.com (S.Z.)

Texts S1. Chemicals and materials.

All VAs were purchased from Dr. Ehrenstorfer Gmbh (Augsburg, Germany). Standard stock solutions (1 mg/mL) of SAs, MAs and TCs were prepared by dissolving 10 mg of the individual compounds in 10 mL of methanol. QAs were dissolved in formic acid and diluted to a final volume of 10 mL with methanol. LAs were prepared in acetonitrile and pure water (1:3, v:v). TCs, SAs, QAs and MAs standard solutions were stored in amber volumetric flasks at -20 °C, which were preferably used within 6 months. The LAs standard solutions were stored in amber volumetric flasks at 4 °C, which were best to be used within a month.

Acetonitrile (ACN), methanol (MeOH) and formic acid (HPLC grade) were purchased from ROE Scientific, Inc. (Newark, USA). Disodium ethylenediaminetetraacetate (Na_2-EDTA) was purchased from Xilong Chemical Co., Ltd. (Guangdong, China). N-EVAP 112 nitrogen evaporator with water bath and gas nozzles was obtained from Oganomation Associates Inc, USA. Oasis HLB (6 mL/500 mg) and PRiME HLB (6 mL/200 mg) extraction cartridges were supplied by Waters (Milford, MA, USA). Syringe filters were purchased from Jinteng Company, Tianjin, China.

Texts S2. Sample preparation for analysis of antibiotic content.

For the original solid samples, they were firstly freeze-dried, followed by being grinded evenly. Then the solid samples (1.0 g for manure, 5.0 g for soil and feed) were put into 50 mL centrifuge tubes. MeOH, CAN and citric acid buffer solution were used as extraction solvents.10 mL mixed solution of MeOH: ACN: citrate buffer (1:1:2, v:v:v) was put into the above mentioned 50 mL centrifuge tubes to extract, followed by vortex for 3 min, ultrasound for 10 min and centrifuge at 10000 r/min for 15 min (4 °C). Then the supernatant was collected in a beaker. Then the above extraction processes were repeated 2 times. All the supernatant was collected and diluted to 200 mL with deionized water. Then the samples were passed through waters Oasis HLB cartridges at a flow rate lower than 3 mL/min. Before sample loading, the Oasis HLB cartridges were preconditioned according to their operating requirements: they were activated with 6 mL of methanol

and 6 mL of deionized water at a flow rate lower than 3 mL/min. After samples loading, the cartridges were dried for 10 min under vacuum, and then were eluted with 6 mL of methanol. Then the eluent was blown to nearly dry by nitrogen at temperature below 40 °C. Then the obtained substances were subsequently dissolved in a mixture of MeOH/water (1:1, v:v) containing 1% formic acid, to a final volume of 1.0 mL. After filtration with 0.22 μm nylon syringe filters, the final extracts were stored in amber glass vials at -20 °C until analysis.

For liquid samples, the volume for most of the wastewater samples was 50 mL. Before extraction, 0.1 g $Na_2EDTA \cdot 2H_2O$ was added to the samples as a chelating agent to reduce antibiotics binding to cations. Then the pH of samples was adjusted to around 3.0 with 1.5 M H_2SO_4, and centrifuged at 10000 r/min for 15 min (4 °C). The samples were then passed through the PRiME HLB cartridges at a flow rate of around 10 mL/min. After that, the HLB cartridges were rinsed with 6 mL ultrapure water to remove residual EDTA, and vacuum dried for 20 min. Finally, the HLB cartridges were eluted with 6 mL of a mixed liquor of MeOH: ACN (1:9, v:v). The extracts were blown to nearly dry by nitrogen at temperature below 40 °C. Then the obtained substances were subsequently dissolved with 1 mL of 0.1% formic acid: methanol (1:1, v:v). After filtration with 0.22 μm nylon syringe filters, the final extracts were stored in amber glass vials at -20 °C until analysis.

Table S1. Basic information of pig farms of different sizes.

Sampling site	Population of pigs	Area covered	The way manure treated
Small-scale pig farm	300 heads	2666.67 m^2	Dry cleaning, compost
Medium-scale pig farm	1200 heads	4000 m^2	Dry cleaning, compost
Large-scale pig farm	30000 heads	66666.7 m^2	Dry cleaning, compost

Table S2. Toxicity data of antibiotics corresponding to sensitive species (wastewater).

Antibiotics	Sensitive species	Toxicity types	AF	Toxicity data (μg/L)	$PNEC_{water}$ (μg/L)	References
SMT	*Microcystis aeruginosa*	acute	1000	$L(E)C_{50}$=23300	23.30	(Isidori et al., 2005)
CIP	*Vibrio fischeri*	acute	1000	$L(E)C_{50}$=2856000	2856	(Halling-Sorensen et al., 2000b)
SMM	*Chlorella vulgaris*	acute	1000	$L(E)C_{50}$=5900	5.9	(Huang et al., 2014)
OFL	*Vibrio fischeri*	acute	1000	$L(E)C_{50}$=13.59	0.01	(Backhaus et al., 2000)
FLU	*Vibrio fischeri*	acute	1000	$L(E)C_{50}$=19.02	0.02	(Backhaus et al., 2000)
PENG	*Microcystis aeruginosa*	acute	1000	$L(E)C_{50}$=6	0.01	(Halling-Srensen et al., 2000a)
LOM	*Vibrio fischeri*	acute	1000	$L(E)C_{50}$=22	0.02	(Robinson et al., 2005)
ENR	*Vibrio fischeri*	acute	1000	$L(E)C_{50}$=49	0.05	(Backhaus et al., 2000)
OXO	*Vibrio fischeri*	acute	1000	$L(E)C_{50}$=22.91	0.02	(Backhaus et al., 2000)
NOR	*Vibrio fischeri*	acute	1000	$L(E)C_{50}$=22	0.02	(Backhaus et al., 2000)
OTC	*Microcystis aeruginosa*	acute	1000	$L(E)C_{50}$=207	0.21	(Backhaus et al., 2000)
TC	*Microcystis aeruginosa*	acute	1000	$L(E)C_{50}$=90	0.09	(Lanzkyet al., 1997)

Table S3. Toxicity data of antibiotics corresponding to sensitive species (soil).

Antibiotics	Sensitive species	Toxicity types	AF	Toxicity data (μg/L)	$PNEC_{soil}$ (μg/kg)	References
OFL	*Vibrio fischeri*	acute	1000	$L(E)C_{50}$=100000	31000	(Isidori et al., 2005)
CIP	*Vibrio fischeri*	acute	1000	$L(E)C_{50}$=2856000	1190952	(Halling-Sorensen et al., 2000b)
SPD	Plants	acute	1000	$L(E)C_{50}$=72000	72	(Zhou et al., 2020)
ENR	*Vibrio fischeri*	acute	1000	$L(E)C_{50}$=49	24	(Backhaus et al., 2000)
OTC	*Microcystis aeruginosa*	acute	1000	$L(E)C_{50}$=207	226.3	(Backhaus et al., 2000)
TC	*Microcystis aeruginosa*	acute	1000	$L(E)C_{50}$=90	567.9	(Halling-Srensen et al., 2000a)
LOM	*Vibrio fischeri*	acute	1000	$L(E)C_{50}$=22	93.85	(Robinson et al., 2005)

L(E)C50: lowest median effective concentration value obtained from existing studies.

AF: the evaluation factor.

PNEC: the predicted no-effect concentration of antibiotics.

References

1. Backhaus, T.; Scholze, M.; Grimme, L.H. The single substance and mixture toxicity of quinolones to the bioluminescent bacterium *Vibrio fischeri*. *Aquat. Toxicol*. **2000**, *49*, 49–61.
2. Halling-Sørensen, B. Algal toxicity of antibacterial agent Used in intensive farming. *Chemosphere* **2000**, *40*, 731–739.
3. Halling-Sørensen, B.; Holten Lützhøft, H.-C.; Andersen, H.R.; Ingerslev, F. Environmental risk assessment of antibiotics: Comparison of mecillinam, trimethoprim and ciprofloxacin. *J. Antimicrob. Chemother*. **2000**, *46*, 53–58.
4. Huang, D.J.; Hou, J.H.; Kuo, T.F.; Lai, H.T. Toxicity of the veterinary sulfonamide antibiotic sulfamonomethoxine to five aquatic organisms. *Environ. Toxicol. Pharmacol.* **2014**, *38*, 874–880.
5. Isidori, M.; Lavorgna, M.; Nardelli, A.; Pascarella, L.; Parrella, A. Toxic and genotoxic evaluation of six antibiotics on non-target organisms. *Sci. Total Environ*. **2005**, *346*, 87–98.
6. Lanzky, P.F.; Halting-Sørensen, B. The toxic effect of the antibiotic metronidazole on aquatic organisms. *Chemosphere* **1997**, *35*, 2553–2561.
7. Robinson A A, Belden J B, Lydy M J. Toxicity of fluoroquinolone antibiotics to aquatic organisms. *Environ. Toxicol. Chem*. **2005**, *24*, 423–430.
8. Zhou, X.; Wang, J.; Lu, C.; Liao, Q.; Gudda, F.O.; Ling, W. Antibiotics in animal manure and manure-based fertilizers: Occurrence and ecological risk assessment. *Chemosphere* **2020**, *255*, 127006.

Table S4. The other 27 antibiotics concentration in manure in spring, summer, autumn and winter (mg/kg).

Antibiotics		Spring (N=60)			Summer (N=60)			Autumn (N=60)			Winter (N=60)		
Class	Type	Min	Max	Mean	Min	Max	Mean	Min	Max	Mean	Min	Max	Mean
QAs	ENR	ND	0.11	0.06	ND	0.34	0.11	ND	0.06	0.05	ND	0.55	0.10
	DIF	ND	0.06	0.06	ND	0.09	0.04	ND	0.05	0.05	ND	0.05	0.05
	SPA	ND	0.03	0.02	ND	0.01	0.00	ND	0.03	0.02	ND	0.03	0.02
	NAL	ND	ND	ND	ND	ND	ND	ND	ND	ND	ND	ND	ND
	FLE	ND	0.08	0.07	ND	0.02	0.02	ND	0.08	0.06	ND	0.09	0.07
	ENO	ND	0.11	0.09	ND	0.08	0.04	ND	0.09	0.07	ND	0.10	0.08
	CIN	ND	0.07	0.06	ND	0.49	0.15	ND	0.02	0.02	ND	0.02	0.02
	ORB	ND	0.07	0.03	ND	0.09	0.02	ND	0.05	0.03	ND	0.05	0.04
	OXO	ND	0.03	0.01	ND	0.01	0.01	ND	0.02	0.02	ND	0.02	0.02
	NOR	ND	0.06	0.05	ND	0.15	0.05	ND	0.07	0.06	ND	0.08	0.06
SAs	SIM	ND	ND	ND	ND	ND	ND	ND	ND	ND	ND	ND	ND
	SDZ	ND	0.01	0.01	ND	0.01	0.00	ND	ND	ND	ND	0.01	0.00
	STZ	ND	ND	ND	ND	0.01	0.00	ND	ND	ND	ND	0.00	0.00
	SMX1	ND	0.03	0.03	ND	0.04	0.01	ND	0.00	0.00	ND	0.00	0.00
	SPD	ND	ND	ND	ND	0.00	0.00	ND	0.01	0.00	ND	0.01	0.00
	SMR	ND	0.02	0.01	ND	0.00	0.00	ND	0.01	0.00	ND	0.01	0.00
	SMT	ND	0.03	0.02	ND	0.03	0.01	ND	0.00	0.00	ND	0.00	0.00
	SDX	ND	0.02	0.02	ND	ND	ND	ND	0.00	0.00	ND	0.00	0.00
	SMX2	ND	0.03	0.03	ND	0.00	0.00	ND	0.00	0.00	ND	0.01	0.01
	SIX	ND	0.02	0.02	ND	0.00	0.00	ND	ND	ND	ND	0.00	0.00
	SB	ND	ND	ND	ND	ND	ND	ND	ND	ND	ND	ND	ND
	SDM	ND	0.01	0.01	ND	0.00	0.00	ND	0.00	0.00	ND	0.00	0.00
	SQX	ND	0.01	0.01	ND	0.01	0.00	ND	0.00	0.00	ND	0.00	0.00
MAs	RTM	ND	0.01	0.01	ND	0.00	0.00	ND	0.01	0.01	ND	0.01	0.01
	CLA	ND	0.00	0.00	ND	0.04	0.01	ND	0.02	0.02	ND	0.02	0.02
	SPI	ND	0.04	0.01	ND	0.06	0.02	ND	0.02	0.02	ND	0.06	0.02
LAs	OXA	ND	0.01	0.01	ND	ND	ND	ND	ND	ND	ND	0.00	0.00

ND: not detected; N: number of samples analyzed; Max: maximum concentration (mg/kg); Min: minimum concentration (mg/kg); 0.00: < 0.005 mg/kg

Table S5. The other 27 antibiotics concentration in wastewater in spring, summer, autumn and winter (μg/L).

Antibiotics		Spring (N=30)			Summer (N=30)			Autumn (N=30)			Winter (N=30)		
**Class**	**Type**	**Min**	**Max**	**Mean**	**Min**	**Max**	**Mean**	**Min**	**Max**	**Mean**	**Min**	**Max**	**Mean**
QAs	ENR	ND	1.39	1.34	ND	ND	ND	ND	1.20	1.15	ND	1.42	1.24
	DIF	ND	0.73	0.71	ND	1.05	0.84	ND	0.53	0.53	ND	0.70	0.59
	SPA	ND	0.82	0.74	ND	ND	ND	ND	0.83	0.82	ND	0.94	0.90
	NAL	ND	1.29	1.26	ND	0.71	0.68	ND	0.92	0.92	ND	0.96	0.95
	FLE	ND	ND	ND	ND	ND	ND	ND	ND	ND	ND	0.54	0.51
	ENO	ND	0.62	0.57	ND	ND	ND	ND	0.54	0.53	ND	0.89	0.70
	CIN	ND	2.41	1.75	ND	ND	ND	ND	ND	ND	ND	ND	ND
	ORB	ND	0.62	0.58	ND	0.51	0.51	ND	ND	ND	ND	0.76	0.74
	OXO	ND	2.12	1.96	ND	0.86	0.75	ND	1.36	1.34	ND	1.53	1.45
	NOR	ND	1.10	1.02	ND	1.38	1.00	ND	1.03	1.00	ND	1.66	1.66
SAs	SIM	ND	ND	ND	ND	ND	ND	ND	ND	ND	ND	ND	ND
	SDZ	ND	1.07	0.80	ND	2.25	1.47	ND	ND	ND	ND	ND	ND
	STZ	ND	0.27	0.27	ND	ND	ND	ND	ND	ND	ND	ND	ND
	SMX1	ND	0.60	0.59	ND	0.36	0.22	ND	ND	ND	ND	0.18	0.18
	SPD	ND	ND	ND	ND	ND	ND	ND	ND	ND	ND	ND	ND
	SMR	ND	ND	ND	ND	ND	ND	ND	ND	ND	ND	ND	ND
	SMM	ND	22.33	5.89	ND	15.88	6.04	ND	21.74	13.57	ND	123.33	78.96
	SMT	ND	0.42	0.37	ND	ND	ND	ND	ND	ND	ND	ND	ND
	SDX	ND	0.41	0.41	ND	ND	ND	ND	ND	ND	ND	ND	ND
	SMX2	ND	0.59	0.55	ND	0.10	0.10	ND	ND	ND	ND	0.12	0.11
	SIX	ND	0.33	0.33	ND	ND	ND	ND	ND	ND	ND	ND	ND
	SB	ND	0.14	0.14	ND	ND	ND	ND	ND	ND	ND	ND	ND
	SDM	ND	0.17	0.16	ND	ND	ND	ND	0.41	0.28	ND	1.07	1.04

	SQX	ND	0.17	0.16	ND	ND	ND	ND	ND	ND	ND	ND	ND
MAs	RTM	ND	0.10	0.10	ND	0.20	0.17	ND	0.28	0.28	ND	0.28	0.28
	CLA	ND	ND	ND	ND	0.19	0.19	ND	0.44	0.44	ND	0.44	0.44
	SPI	ND	ND	ND	ND	0.29	0.20	ND	0.38	0.36	ND	0.47	0.43
LAs	OXA	ND	0.23	0.20	ND	ND	ND	ND	ND	ND	ND	ND	ND

ND: not detected; N: number of samples analyzed; Max: maximum concentration (mg/kg); Min: minimum concentration (mg/kg); 0.00: < 0.005 mg/kg

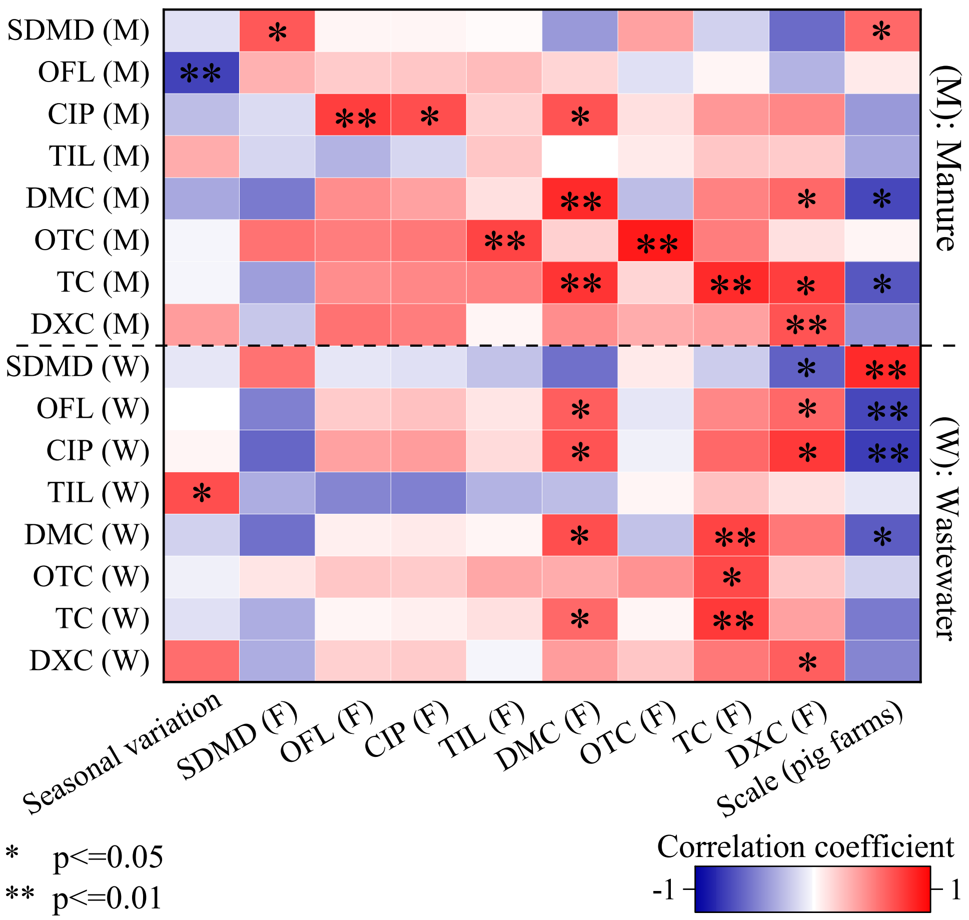

Figure S1. The potential effects of seasonal variation, feed and pig farm size on antibiotic residues in pig manure and wastewater (F means feed; W means wastewater; M means manure).
